# COBREXA 2: tidy and scalable construction of complex metabolic models

**DOI:** 10.1093/bioinformatics/btaf056

**Published:** 2025-02-08

**Authors:** Miroslav Kratochvíl, St Elmo Wilken, Oliver Ebenhöh, Reinhard Schneider, Venkata P Satagopam

**Affiliations:** Luxembourg Centre for Systems Biomedicine, University of Luxembourg, Esch-sur-Alzette L-4362, Luxembourg; Institute of Quantitative and Theoretical Biology, Heinrich Heine University, Düsseldorf, North Rhine-Westphalia 40225, Germany; Cluster of Excellence on Plant Sciences, Heinrich Heine University, Düsseldorf, North Rhine-Westphalia 40225, Germany; Institute of Quantitative and Theoretical Biology, Heinrich Heine University, Düsseldorf, North Rhine-Westphalia 40225, Germany; Cluster of Excellence on Plant Sciences, Heinrich Heine University, Düsseldorf, North Rhine-Westphalia 40225, Germany; Luxembourg Centre for Systems Biomedicine, University of Luxembourg, Esch-sur-Alzette L-4362, Luxembourg; Luxembourg Centre for Systems Biomedicine, University of Luxembourg, Esch-sur-Alzette L-4362, Luxembourg

## Abstract

**Summary:**

Constraint-based metabolic models offer a scalable framework to investigate biological systems using optimality principles. Construction and simulation of detailed models that utilize multiple kinds of constraint systems pose a significant coding overhead, complicating implementation of new types of analyses. We present an improved version of the constraint-based metabolic modeling package COBREXA, which utilizes a hierarchical model construction framework that decouples the implemented analysis algorithms into independent, yet re-combinable, building blocks. By removing the need to re-implement modeling components, assembly of complex metabolic models is simplified, which we demonstrate on use-cases of resource-balanced models, and enzyme-constrained flux balance models of interacting bacterial communities. Notably, these models show improved predictive capabilities in both monoculture and community settings. In perspective, the re-usable model-building components in COBREXA 2 provide a sustainable way to handle increasingly complex models in constraint-based modeling.

**Availability and Implementation:**

COBREXA 2 is available from https://github.com/COBREXA/COBREXA.jl, and from Julia package repositories. COBREXA 2 works on all major operating systems and computer architectures. Documentation is available at https://cobrexa.github.io/COBREXA.jl/.

## 1 Background 

Constraint-based reconstruction and analysis (COBRA) tools seek to predict cellular physiology from bottom-up reconstructed metabolic models. These models are constructed with limited experimental measurements yet can be used to understand biologically important metabolic phenomena, such as overflow metabolism. This dramatically reduces the difficulty of investigating complex systems that range from single bacteria to whole humans and their associated microbiomes (Monk *et al.* 2017, Heinken *et al.* 2023). COBRA models are typically simulated using Flux Balance Analysis (FBA), which assumes metabolic steady state across all reactions and satisfaction of some optimality metric (Orth *et al.* 2010), usually encoded in a linear constrained optimization problem. Importantly, FBA is extensible, and increasingly realistic models may be built by introducing additional constraints, such as enzyme kinetics, proteome capacity limitations, translation costs, and resource parsimony. Further, FBA extends to multi-compartment systems, allowing simulation of microbial communities (Nilsson *et al.* 2017), often including additional constraints and more complex objective functions (Kim *et al.* 2022).

Many algorithms have been implemented to simulate individual FBA extensions (such as enzyme-constrained and community models) in isolation. However, running simulations of new extensions, or mere combinations of existing ones, often poses major programming effort. For example, algorithms for parallel evaluation of biomass production envelopes, community flux balance modeling, and resource-balance analysis (RBA) are all available in current software (Ebrahim *et al.* 2013, Bulović *et al.* 2019, Diener *et al.* 2020), but running these extensions in combination is not supported.

Here, we present an improved version of COBREXA which, atop of the horizontal scalability retained from the original version (Kratochvíl *et al.* 2022), provides a novel systematic approach that addresses the re-implementation overhead associated with analysis extensions. Specifically, we introduce *constraint trees* as an intermediate layer for assembling and extending the constraint systems. Constraint trees organize the constraints hierarchically into directory-like structures that may be freely explored and arbitrarily combined via a small set of well-defined methods with predictable behavior. In a manner similar to “tidy” data manipulation (Wickham 2014), this helps to reduce the coding overhead imposed on the users: because constraint trees provide a lightweight and universal unifying layer for all implemented constraint systems and analysis methods, the programming effort that commonly arises from repurposing analysis algorithms and combining unstructured constraint-system representations is effectively prevented.

We showcase this approach by implementing community-scale enzyme constrained FBA models of interacting *Escherichia coli* mutants, as well as a simplified variant of resource balance analysis that incorporates ribosome utilization across different growth rates for *E. coli*. Importantly, these models were incrementally constructed from a base FBA model, by successively accumulating small repurposable “building-blocks” represented as constraint trees, allowing substantial reduction of the coding effort (see Supplementary Section S1.1 for details). Results obtained from the models indicate that such extensions to COBRA methods improve alignment with experimental observations, suggesting that a streamlined, modular framework for extending metabolic models will become more important in the future of the field.

## 2 Implementation

Briefly, constraint trees represent a set of labeled constraints organized in a directory structure, each of which binds a value (an expression over variables, typically a linear combination thereof) to a bound (such as equality or interval bound). This design combines both the user-friendly way of accessing and adjusting parts of the model as sub-objects via the familiar dot notation, as common in object-oriented systems (such as COBRApy; Ebrahim *et al.* 2013), and the generality of matrix-based representations of constraint systems that impose no hard-coded semantics, guaranteeing that the systems can be extended to any constraint-based modeling problem at hand. In turn, this combination alleviates common issues in extension implementations, such as index calculations and identifier mangling. Details are provided in Supplementary Section S4.

**Figure 1.**
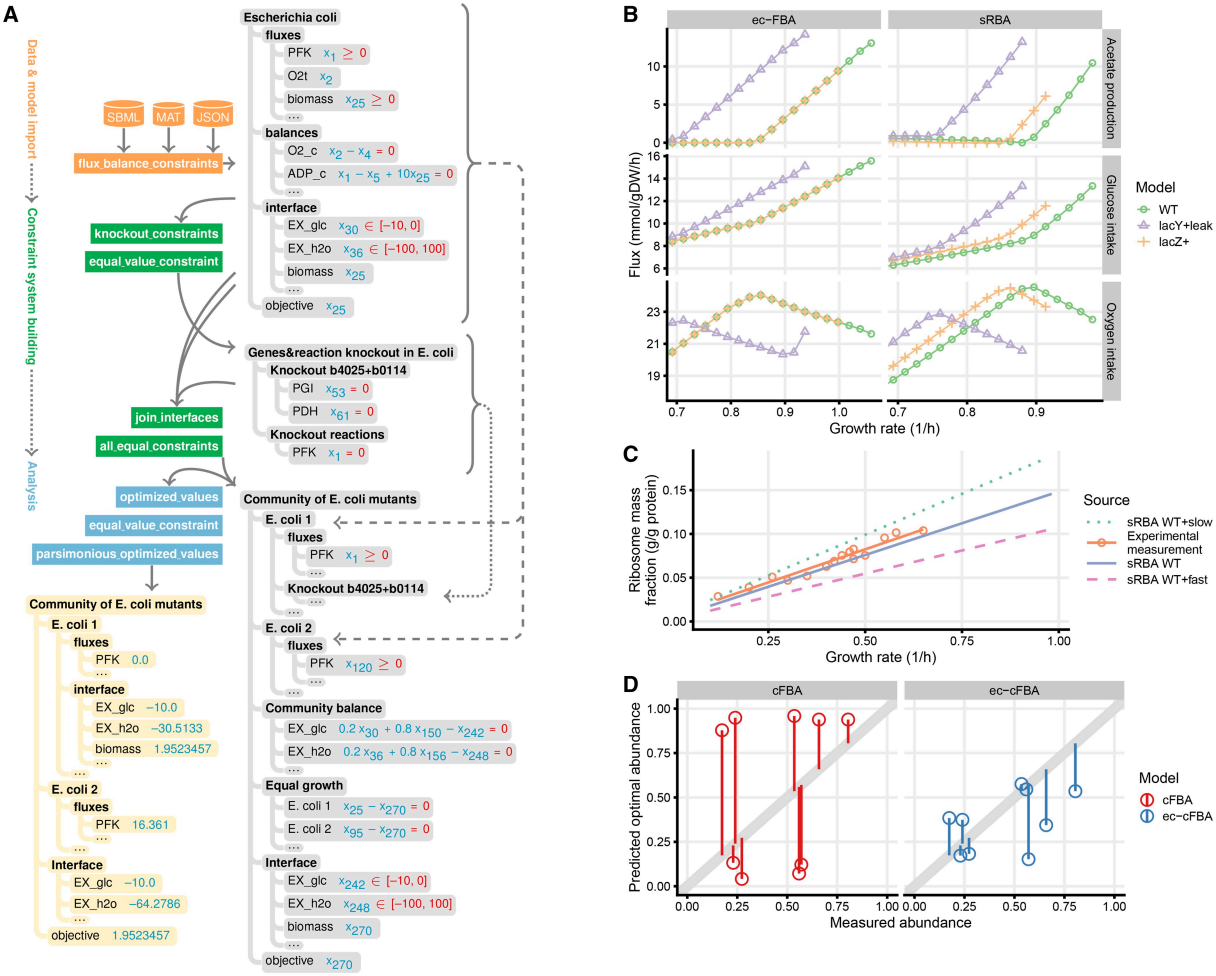
(A) Simplified example of using constraint trees (in gray) in COBREXA 2 for representing intermediate model parts, used to run a parsimonious community FBA. (B) sRBA model constructed with COBREXA 2 predicts earlier onset of overflow metabolism under over-expression of proton-leaking lacY and lacZ, improving the consistency with experimental results (Basan *et al.* 2015) over ec-FBA. (C) The dependence of ribosome-to-protein mass ratio on growth rate computed by sRBA parametrized by average (sRBA WT) and minimal/maximal observed (sRBA WT+slow and +fast) translation rate, compared to experimental measurements (Hu *et al.* 2020). (D) Community growth-rate-maximizing composition predictions produced by ec-cFBA reflect experimental data better than conventional cFBA.

COBREXA 2 is implemented as a metabolic modeling front-end for constraint trees. A typical workflow is sketched in Fig. 1A: Input data (reaction stoichiometry system, model parameters, etc.) are first interpreted as constraint tree structures. Individual constraint trees and their parts are re-used and combined, creating a successively more complex system—e.g. whole sub-trees may be copied to create multiple versions of model compartments; and individual values may be picked by name and re-constrained to implement knockouts. The system is finally passed to an optimizer for solving; the results are presented to user via the same structure as the original constraint tree, improving transparency.

COBREXA 2 provides two informal layers of functions to facilitate such workflows: the reconstruction layer builds constraint trees from the available base data, including the models and their compartments, as well as estimated and experimentally measured parameters. This allows users to construct a constraint system that clearly describes each facet of the model, such as mass balances, metabolite exchanges, knockouts, community dynamics, enzyme kinetics, and resource parsimony constraints. The analysis layer implements algorithms to scrutinize the system, such as FBA, flux variability analysis, flux sampling, and production envelopes. Functionality of both layers is exposed to users, enabling any components to be directly combined.

## 3 Selected use-cases

### 3.1 Resource allocation constraints improve prediction of overflow metabolism onset

To demonstrate incremental building of complex models in COBREXA 2, we show layered construction of a resource-balanced model based on the iML1515 model of *E. coli*: first, we added enzyme kinetics, as well as both cytosolic and membrane capacity constraints to the model, yielding an enzyme-constrained FBA (ec-FBA) model. Second, we extended this enzyme constrained model further, by incrementally adding ribosome kinetics, utilization, and capacity constraints; protein polymerization ATP-cost constraints; as well as incorporating growth dilution of biomass components. This yielded a simplified resource balance analysis (sRBA) model (see Supplementary Section S2.1 for all details). Thanks to the provided abstractions, the code that assembles the sRBA constraints from prepared data is remarkably succinct, totaling only 90 lines of commented Julia.

We observed that while ec-FBA successfully models the onset of overflow metabolism, it failed to predict its earlier onset when a “useless” (in the context of glucose-driven growth) cytosolic protein, lacZ, is over-expressed, contradicting experimental results (Basan *et al.* 2015; Fig. 1B). This misprediction was rectified by the sRBA model, which correctly predicted the earlier onset of overflow metabolism when either a proton leaking membrane protein, lacY, or lacZ are over-expressed, as shown in Fig. 1B. Encouragingly, we also observed a good correlation of the ribosome-to-protein mass fractions obtained from the sRBA simulation to experimental measurements (Dai *et al.* 2016; Fig. 1C), suggesting that sRBA captures mechanistically important model properties.

### 3.2 Enzyme constraints improve community FBA growth predictions

Since the ec-FBA formulation improves predictions for single organism models (Domenzain *et al.* 2022), it is natural to ask if similar benefits could be obtained from enzyme constraints in community FBA (cFBA) models (Khandelwal *et al.* 2013). We used COBREXA 2 to construct enzyme-constrained cFBA (ec-cFBA) models of auxotrophic *E. coli* mutants, and compared the predictions of optimally-growing community compositions to plain cFBA, as well as experimental data (Mee *et al.* 2014; see Supplementary Section S2.3 for details). Notably, these constructions were made feasible at the community scale only by recent advances in *in silico* estimation and optimization of enzyme kinetic parameters (Wilken *et al.* 2022, Kroll *et al.* 2023).

We observed that ec-cFBA simulations of mutually interdependent auxotrophic *E. coli* co-cultures clearly show two different growth regimes and a well-defined growth optimum—the growth rate was highly dependent on the community composition, as opposed to plain cFBA where the community composition only caused a negligible effect (Supplementary Fig. S4). When we extended the same analysis to all co-culture communities that demonstrated significant experimental growth (Mee *et al.* 2014), ec-cFBA models provided substantially better predictions over cFBA (Fig. 1D), improving the correlation with experimental data from 0.197 (cFBA) to 0.453 (ec-cFBA).

## 4 Discussion

Resource allocation constraints are increasingly used in metabolic modeling, due to their ability to mechanistically describe complex metabolic phenomena. We showcased that even a simplified RBA model recapitulates the experimental findings of *E. coli* metabolism better compared to an enzyme-constrained model: building on theoretical developments, we included both membrane and cytosolic protein capacity constraints to model the onset of overflow metabolism (Zhuang *et al.* 2011, De Groot *et al.* 2020). Interestingly, both the ec-FBA and sRBA models asserted higher sensitivity of the onset to membrane capacity limitations (Supplementary Section S2.1).

In the community setting, our results suggest that enzyme capacity plays an important role in determining steady-state community abundances. However, simulations of a 4-member community of *E. coli* mutants still showed a substantial compositional variability of near-optimal growth rates (Supplementary Fig. S5), which is contradicted by experimental results (Mee *et al.* 2014), suggesting existence of additional significant driving forces behind the community dynamics. We expect that substantially more complex models will be required to provide sufficient predictive power for such communities.

We demonstrated that COBREXA 2 and the constraint trees framework enable rapid implementation of advanced modeling approaches, like community-scale resource-constrained models. In the future, we hope that the model construction approach introduced by COBREXA 2 will aid further refinement of such models with new kinds of constraint systems, enabling further improvements of the model predictive abilities and thus deeper investigation of the biological mechanisms at the root of complex metabolic phenomena.

## Supplementary Material

btaf056_Supplementary_Data

## Data Availability

Data and scripts that replicate the results are available from https://doi.org/10.5281/zenodo.14606532.
