# Supplementary material for: COBREXA 2: tidy and scalable construction of complex metabolic models
Source: Bioinformatics. 2025 Feb 8;41(2):btaf056. doi: 10.1093/bioinformatics/btaf056 (PMC11842047; doi:10.1093/bioinformatics/btaf056)
Supplement: btaf056_Supplementary_Data [file btaf056_supplementary_data.pdf]

Supplementary information for: COBREXA 2

Authors of COBREXA 2

S1 Elaborated constraint systems

S1.1 Incremental construction of resource-balanced models

Enzyme-balanced FBA construction

FBA models are composed of a stoichiometric matrix, $\mathbf{S}$, enforcing mass balance on a vector of the modeled fluxes, v, which are usually directionally constrained through lower bounds $\mathbf{v}_{\text{LB}}$ and upper bounds $\mathbf{v}_{\text{UB}}$. A typical FBA representation is shown in Supplementary System 1: The system is formulated as an optimization problem that maximizes the biomass objective function (μ); the optimum is then the highest-yield solution [1].

Enzyme kinetics computation requires a 'unidirectional' view of the reactions in the model; we therefore split the (possibly bidirectional) variables in the model into their unidirectional counterparts using Supplementary System 2.

Enzyme-constrained FBA (ec-FBA) models extend Supplementary System 2 with enzyme kinetics, derived mainly from turnover numbers (k_{cat}s) and proteome capacity limitations [2, 3, 4]. ec-FBA models explicitly account for the protein cost, associated with a metabolic flux. A complete ec-FBA formulation is shown as Supplementary System 3.

In Supplementary System 3 we explicitly make room multiple capacity bounds $E_{1,\ldots,n}$ where the enzymes are selected and weighted by corresponding vectors $m_{1,\ldots,n}$.

Simplified RBA

Resource balance analysis (RBA) seeks to extend ec-FBA models by incorporating gene expression (transcription and translation) into the model formulation [5, 6]. Simplified RBA (sRBA), as presented in Supplementary System 5 partially shares this goal, but only accounts for translation (ribosomes), energy costs associated with protein synthesis, and biomass component growth dilution.

Since we are interested in a chemostat-like simulation, we fix μ and minimize the resource parsimony objective: $\sum_j e_j \cdot m_j + m_k \cdot \sum_k r_k$ (which is equivalent to minimizing the L1 norm of the protein content of the model).

Implementation of Supplementary System 5 in COBREXA 2 is available in the supplementary code archive at `https://doi.org/10.5281/zenodo.14606532`, file `scripts/03_srba.jl`, function `with_srba_constraints`, totaling 90 lines of commented code.

A usecase-independent implementation of sRBA is available as a reusable example notebook in COBREXA.jl documentation, or via `https://cobrexa.github.io/COBREXA.jl/v2.7.0/examples/07a-srba/`.

$$
\begin{aligned}
\max \mu & & \text{maximize growth} \\
\mu = \mathbf{v}^\mathsf{T}\mathbf{c} & & \text{objective function} \\
\mathbf{S} \cdot \mathbf{v} = \mathbf{0} & & \text{mass balance} \\
\mathbf{v}_{\mathrm{LB}} \leq \mathbf{v} & & \text{lower flux bounds} \\
\mathbf{v}_{\mathrm{UB}} \geq \mathbf{v} & & \text{upper flux bounds}
\end{aligned}
$$

Supplementary System 1: Flux balance analysis as a linear program. The variables in this program are the fluxes, $\mathbf{v}$ and the biomass objective function, μ. In COBREXA, this system is generated via `flux_balance_constraints`.

$$
\begin{aligned}
\mathbf{u}^{+} \geq 0 & & \text{forward fluxes} \\
\mathbf{u}^{-} \geq 0 & & \text{reverse fluxes} \\
\mathbf{u}^{+} \leq \mathbf{v}_{\mathrm{UB}} & & \text{forward flux bounds} \\
\mathbf{u}^{-} \leq -\mathbf{v}_{\mathrm{LB}} & & \text{reverse flux bounds} \\
\mathbf{u}^{+} - \mathbf{u}^{-} = \mathbf{v} & & \text{directionality balance}
\end{aligned}
$$

Supplementary System 2: Unidirectional reaction variable system as an extension of Supplementary System 1. The additional variables here are the unidirectional fluxes in the forward, and reverse directions, $\mathbf{u}^{+}$, $\mathbf{u}^{-}$. Notably, the system allows the reactions to 'run' in both forward and reverse direction at once. In COBREXA, this system is generated via `sign_split_constraints` and several related functions.

$$
\begin{aligned}
\mathbf{e} \geq 0 & & & \text{isozyme amounts} \\
\sum_{i \in \textsc{FwdIsos}(r)} \mathbf{k}_{\mathrm{cat}\ i} \cdot \mathbf{e}_i \geq |\mathbf{u}_r^{+}| & & (\forall r \in R_e) & \text{forward catalysis capability of isozymes} \\
\sum_{i \in \textsc{RevIsos}(r)} \mathbf{k}_{\mathrm{cat}\ i} \cdot \mathbf{e}_i \geq |\mathbf{u}_r^{-}| & & (\forall r \in R_e) & \text{reverse catalysis capability of isozymes} \\
(\forall i) \quad e^\mathsf{T} m_i \leq E_i & & & \text{capacity limitations}
\end{aligned}
$$

Supplementary System 3: ec-FBA as constraints that extend the FBA with unidirectional reactions (Supplementary System 2). The new variables stand for enzyme concentrations $\mathbf{e}$, and the grouped enzyme capacity bounds E_i (each i thus specifies a group of enzymes to be bounded). In COBREXA, this system is generated via `enzyme_constraints`.

$$
E_{\mathrm{mem}} = 0.2 \cdot \sum_i E_i \qquad \text{membrane-to-total protein mass ratio}
$$

Supplementary System 4: Additional ec-FBA constraint to keep the membrane protein capacity in biologically expectable range. In COBREXA, this system is generated via `equal_value_constraint` and adding and scaling of values in constraint trees. (Index of E_{mem} labels one of the general indices i of E in Supplementary System 3).

$$\max \mu' \qquad \text{maximize resource-based growth}$$

$$\mathbf{0} = \begin{pmatrix} \mathbf{S}' & \mathbf{b} \end{pmatrix} \cdot \begin{pmatrix} \mathbf{v}' \\ \mu' \end{pmatrix} \qquad \text{metabolite and biomass balance}$$

$$\mathbf{b}_i = \begin{cases} -\left(\sum_j e_j \cdot \mathrm{N_i}(e_j) + \sum_k r_k \cdot \mathrm{N_i}(r_k)\right) & \text{if } i \in \text{amino acids} \\ \mathbf{S}_{i,\text{biomass}} + \nu_i \left(\sum_j e_j \cdot \mathrm{N}_*(e_j) + \sum_k r_k \cdot \mathrm{N}_*(r_k)\right) & \text{if } i \in \begin{cases} \text{ATP} \\ \text{ADP} \\ \text{H}_2\text{O} \\ \text{H}^+ \\ \text{Pi} \end{cases} \\ \mathbf{S}_{i,\text{biomass}} & \text{otherwise} \end{cases} \qquad \text{biomass composition}$$

$$\mu' \cdot e_i = \frac{k_r}{\mathrm{N}_*(e_i)} \cdot r_i \quad (\forall i \in \text{proteins}) \qquad \text{enzyme translation}$$

$$\mu' \cdot \sum_i r_i = \frac{k_r}{\mathrm{N}_*(r_r)} \cdot r_r \qquad \text{ribosome translation}$$

$$E_{\text{mem}} \geq \sum_i m_i \cdot e_i \qquad \text{enzyme mass on membrane}$$

$$E_{\text{total}} \geq \sum_i m_i \cdot e_i + \sum_i m_i \cdot r_i \qquad \text{total protein capacity}$$

Supplementary System 5: sRBA as constraints that extend the ec-FBA (Supplementary System 3). In the system, the biomass function decomposition to components ($\mathbf{b}_i$) ensures that non-amino acid and energy metabolites are produced at the same rate as the ec-FBA model, and that the energy and amino-acid cost of the transcription scales with the amount of enzymes required to catalyze the metabolic flux. The total number of amino acids in a molecule x is counted by $\mathsf{N}_*(x)$, and the number of amino acids of type y in a molecule x is counted by $\mathsf{N}_\mathsf{y}(x)$. P is the ATP cost of polymerization of an amino acid into a protein (by default, $P = 4.2$). k_r is the ribosome translation rate is (by default, $k_r = 12$). The ATP requirement for growth is adjusted according to the amount of ATP is needed to produce the enzymes, $\mathbf{e}$, and the ribosomes, $\mathbf{r}$. The ATP hydrolysis equation is used to modify the amount of energy currency metabolites in the original biomass function, $\text{ATP} + \text{H}_2\text{O} \longrightarrow \text{ADP} + \text{PO4} + \text{H}^+$ — here, ν_i marks the stoichiometry of the metabolites in this reaction. Notably, the sRBA model is bilinear in μ and $(\mathbf{e}, \mathbf{r}, \mathbf{v})$. Enough ribosomes must be made to produce all the enzymes, as well as the ribosomes necessary to produce the enzymes.

S1.2 Constraint systems for enzyme-constrained communities

Supplementary Figure S1 is provided as an illustration of the situation in a co-culture of auxotrophic organisms.

ec-cFBA models used in the manuscript are illustrated in Supplementary Figure S1. The formulation of the ec-cFBA problem is listed in Supplementary System 6.

Implementation of Supplementary System 6 in COBREXA 2 is available in the supplementary code archive at `https://doi.org/10.5281/zenodo.14606532`, file `scripts/common/auxotrophe_community.jl`, function `auxotrophe_community`, totaling 71 lines of commented code.

A usecase-independent implementation of ec-cFBA is also available as a reusable example notebook in COBREXA.jl documentation, or via `https://cobrexa.github.io/COBREXA.jl/v2.7.0/examples/07b-community-ecfba/`.

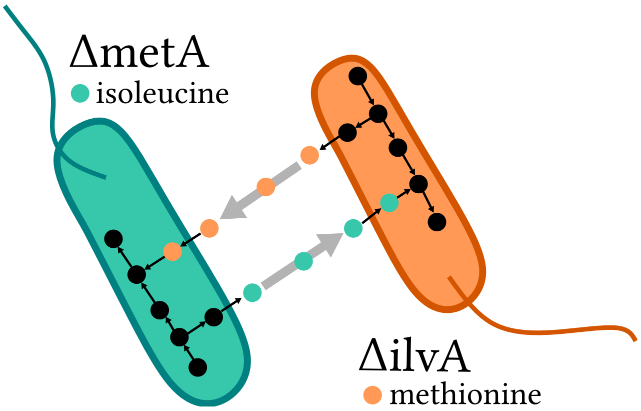

Figure S1: Illustration of mutually auxotrophic *E. coli* co-culture. ΔmetA cannot produce methionine, ΔilvA cannot produce isoleucine. The strains can grow only when co-cultured.

$$
\begin{aligned}
&\max\ c && && \text{maximize the community growth} \\
&\mathbf{a}_i \geq 0 && (\forall i) && \text{individual community member abundances} \\
&\sum_i \mathbf{a}_i = 1 && && \text{abundances sum to 1} \\
&\sum_i \mathbf{v}_{i\,x} a_i = \mathbf{v}_{c,x} && (\forall x \in X) && \text{community exchange balance} \\
&\mu_i = c && (\forall i) && \text{community growth balance}
\end{aligned}
$$

Supplementary System 6: Enzyme-constrained 2-member community FBA (ec-cFBA) as a linear program. The problem includes several instances of Supplementary System 3 with all variables indexed by the community member index i (i.e., stoichiometry of the i-th member is $\mathbf{S}_i$ and the internal flux in i-th member is $\mathbf{v}_i$). For simplicity, we assume that the set of exchange reaction indexes X is the same in all community members; realistic software implementations will instead select the reactions by a pre-established shared identifier scheme. In addition to the variables defined previously, a_i represents the abundance of species i. Environmental exchange is modeled by $v_{c,x}$ for metabolite x. In COBREXA, this system is generated via `interface_constraints`; suitable interfaces are obtained from `flux_balance_constraints`.

S2 Construction and interpretation of use-case models

S2.1 Incremental construction of an iML1515 resource-balanced model

To demonstrate the construction of complex models from simple building blocks, we incrementally constructed an example genome-scale enzyme- and translation-constrained model of *E. coli*. Our construction is based on a conventional FBA model of a mass-balanced reaction system, iML1515 [7]. Initially, we created a system of unidirectional reactions atop the original model, and added constraints that incorporate enzyme turnover numbers (k_{cat}s), and proteome capacity limitations [2, 3], which explicitly account for the protein cost of a given metabolic flux. Crucially, since modeling overflow metabolism requires at least two active bounds [8], we added both a membrane bound, and a total proteome density bound to the model. The exact form of the model is elaborated in Section S1.1. For the parametrization of enzymatic constraints, we used *in silico*-estimated enzyme turnover numbers [9] for all presented experiments, together with a total enzyme capacity bound of $0.55\frac{\mathrm{g}}{\mathrm{g_{DW}}}$, and membrane enzyme capacity bound $0.11\frac{\mathrm{g}}{\mathrm{g_{DW}}}$ (i.e., the amount of membrane enzymes is bounded to 20% of the total enzyme amount).

Because protein synthesis is a major biosynthetic cost in bacteria that outweighs both transcription and translation energy requirements [10], we reasoned that a simplified variant of Resource Balance Analysis (RBA) [5, 6], only taking into account ribosomes, ATP, and amino acid requirements of protein synthesis, would be sufficient to mechanistically model overflow metabolism. Through the text, we label this simplified RBA as sRBA. For sRBA models in this work, we set the ribosome translation rate to 12 amino acids per second, and the polymerization cost to 4.2 ATP per amino acid. Additionally, the L1 norm of proteins and ribosomes is minimized at each growth rate, in order to ensure a sufficiently unique solution from the possibly under-determined constraint system.

In this formulation, sRBA is an straightforward extension of enzyme constrained FBA (ec-FBA) models. We note that the full RBA problem formulation that simulates the transcription and replication machinery may be constructed as another extension of this model. As a major benefit compared to full RBA, the parameters required for sRBA model construction are relatively well-determined and easy to collect from public databases. The exact definition of the sRBA model is provided in Section S1.1.

Discussion of results obtained from iML1515 sRBA model

The protein fraction of cellular dry mass varies by less than 10% across a range of growth conditions (Supplementary Figure S2) [11]. This density constraint has been used to provide a mechanistic explanation for overflow metabolism [12]. In essence, the density limitation forces the cell into a trade-off between devoting resources to catabolism and anabolism: During slower growth regimes, the cells favor higher yield but proteomically costlier respiration (with kinetically slower, bigger enzymes), while at higher growth rates, fermentation metabolism is also used (in the case of *E. coli*, this results in aerobic acetate production). Fermentative ATP generation is kinetically faster, requires smaller enzymes, but ATP yield is lower than from respiration. Additionally, since ribosomes are needed to produce both enzymes *and* the ribosomes themselves, and their translation rate is limited, increased ribosome concentrations are required to support higher growth rates [13, 14]. In turn, this leaves even less space for large enzymes, and forms the basis for the resource partition trade-off observed in bacteria, which leads to overflow metabolism [15].

Expectably, we encode the constant density observation into ec-FBA models via a single constraint that restricts the total proteome density to a chosen constant. Additionally, we use a secondary membrane capacity bound to reflect the physical constraint of limited membrane space. Assuming this bound structure causes the onset of overflow metabolism to be controlled by the membrane capacity bound [17, 18], which is in contrast to simpler resource allocation models that only posit a

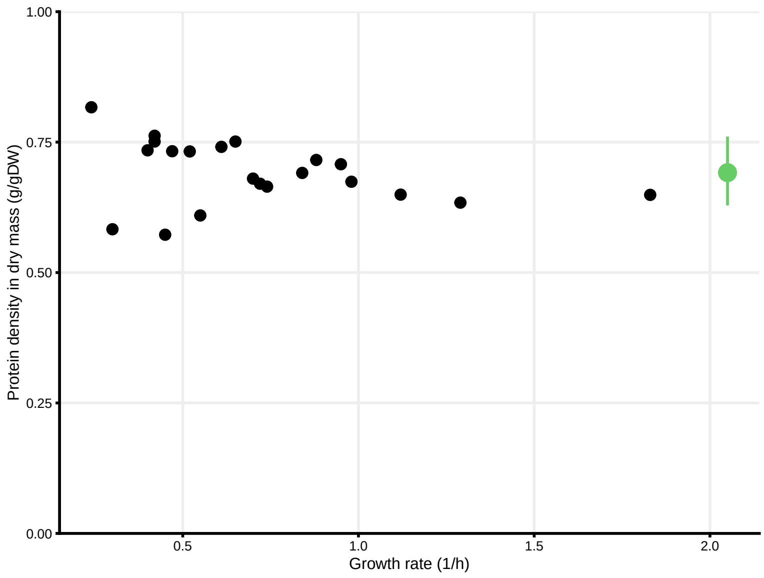

Figure S2: Protein density across a range of culturing conditions of *E. coli*. Dots represent individual experimental measurements [11]. Mean value with 10% relative tolerance is highlighted in green on the right side.

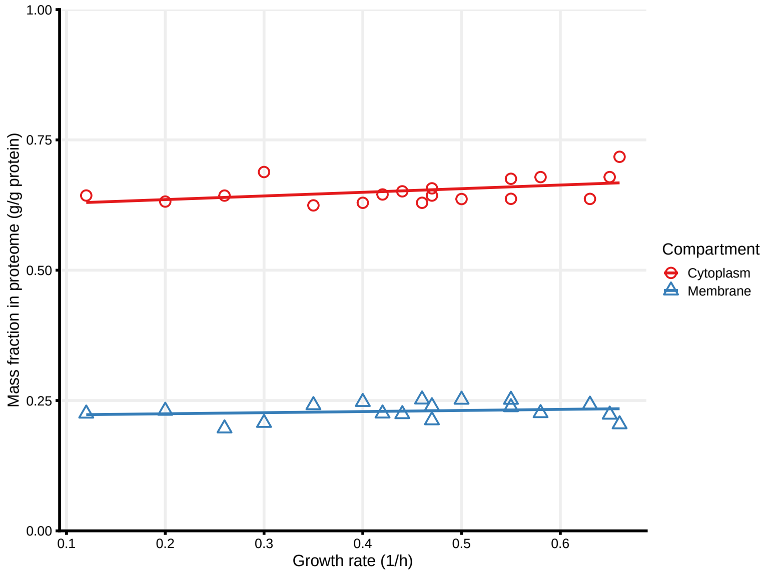

Figure S3: Fraction of cytosolic and membrane-bound proteins measured in different growing conditions of *E. coli*. Points represent experimental measurements [16], lines represent least-squares regression in each compartment. Slope of either of the regression fits is not significantly different from 0.

total capacity bound [2, 19]. This assumption is further supported by recent quantitative proteomics measurements, which revealed the mass ratio between cytosolic and membrane-bound proteins as relatively constant over a wide range of growth conditions (Supplementary Figure S3) [16]. In total, we use these assumptions to set two capacity bounds in the ec-FBA models: a total protein ($0.55\frac{g}{g_{DW}}$), and a membrane capacity bound (20% of the total protein capacity).

Simulations of the constructed *E. coli* ec-FBA model in COBREXA 2 (Supplementary Figure S6) show that when a membrane protein that is 'useless' in the context of glucose-driven growth (e.g. WT lacY or the engineered proton-leaking lacY) is over-expressed, less space remains for transporters and respiratory membrane-bound complexes, causing the earlier onset of overflow metabolism. On the contrary, over-expressing a 'useless' cytosolic protein (lacZ) shows no effect. That contradicts recent experimental results, which showed that over-expressing either of lacZ and the proton-leaking lacY caused the earlier onset of overflow metabolism.

Naturally, we hypothesized that extending the ec-FBA model with further resource allocation constraints would capture the observed phenotypic behavior, and extended the ec-FBA model with sRBA constraints that account for ribosomes that occupy additional cytosolic space, and for the amino-acid and ATP cost of protein polymerization.

The *E. coli* sRBA model improved the predictive accuracy of our simulations over ec-FBA, showing that over-expression of lacZ also leads to the earlier onset of overflow metabolism. We additionally observed that the membrane bound is still a driver of this result, as an over-expression of lacZ requires more ATP to be produced (due to protein polymerization costs), which increases the amount of energy generating membrane proteins at each growth rate relative to the WT simulation. Thus, the earlier onset of the overflow metabolism is caused indirectly by hitting the capacity limitation at the membrane.

Possible interpretations of the results from sRBA simulations

Basan et al. [15] did not find evidence that membrane capacity would determine the switch to the overflow metabolism when attempting to over-express WT lacY, which contradicts our results, where WT lacY causes the switch. At the same time, Wagner et al. [20] found evidence of this effect for several other (GFP-fused, WT) membrane proteins. We hypothesize that this apparent contradiction could be explained by the difficulties of over-expressing membrane proteins.

Further experiments might thus be necessary to elucidate the effect: mainly, the fraction of the proteome taken by the over-expressed lacY should be determined experimentally (these measurements were not reported by Basan et al. [15]). On the other hand, over-expressed lacZ was measured at more than 8% of the proteome, representing substantial metabolic stress (even in terms of ATP polymerization costs). This way, our simulations lend support to the 'membrane real estate hypothesis' [17, 18] for explaining overflow metabolism, but the results are not dispositive.

S2.2 Parameter choices for the sRBA model

To parameterize each example model, we gathered the enzyme turnover numbers directly from [9], the enzyme sub-unit stoichiometry from Uniprot [21] and the Complex Portal [22], and the translation rate from [23].

We opted to use representative values for parameters that have growth rate dependent effects (e.g. translation rate of 12 amino acids per second), and average values specific constants like the ribosome molar mass (2700 kDa[1]), amino acid composition of the ribosome (7459 amino acids per ribosome[2]),

[1] `https://bionumbers.hms.harvard.edu/bionumber.aspx?id=100118&ver=10&trm=e+coli+ribosome+molar+mass&org=`

[2] `https://bionumbers.hms.harvard.edu/bionumber.aspx?id=101175`

ATP protein polymerization cost (4.2 ATP per amino acid[3]), etc. Links to all data sources together with cleaned extracted data files are reported in the source code repository. The parameters were used as gathered, with no parameter fitting procedure involved.

We additionally summarize the sensitivity of the constructed model to the translation rate parameter (k_r), which seemed to be the most sensitive to perturbations: In Figure 1C in main text, we compare of the ribosome mass fractions using the translation rate of 12 amino acids per second (a representative average value) against 9 and 17 amino acids per seconds (the minimum and maximum measured value reported by Dai et al.) [23]. Notably, the average value recapitulates the experimental data well, and the mass fractions seem to differ between the average and extreme parametrization only by a constant relative factor. We did not observe any substantial impact of changing the translation rate on the metabolic flux (Supplementary Figure S6).

S2.3 Construction of enzyme-constrained communities

To further demonstrate the versatility of COBREXA 2, we constructed enzyme-constrained community models of interacting *E. coli* mutants.

These mutants were modeled as auxotrophic for 14 specific amino acids, using single-gene deletions in the relevant biosynthetic pathways, as has been done experimentally [24, 25]. The auxotrophic models were additionally constrained by enzyme kinetic and capacity constraints (analogous to the *E. coli* extension specified in Section S1.1, but only with the total enzyme capacity bound). Individual models were connected via their exchange reactions, with exchange fluxes weighted by the abundance of the mutant, and the biomass production rates of each mutant model was constrained to the same value, effectively creating a community growth rate as in the cFBA method [26]. Additionally, the mutants were allowed to share the knocked-out amino acids with each other. To avoid the bilinearity of the cFBA model (in abundances and fluxes), we solved the problem multiple times over a uniform sample of possible abundances, and picked the solution with the maximum growth rate. The complete constraint system construction is laid out in Section S1.2.

Discussion of results obtained from iML1515 enzyme-constrained auxotrophe community

Thus far, ec-FBA models have been mostly applied to single organisms [27], raising a question: would adding enzyme constraints to community flux balance analysis (cFBA) models improve their predictive accuracy? Previous attempts to answer this question made use of *ad hoc* constraints, e.g. flux balance analysis with molecular crowding (FBAwMC) [28] was incorporated into a community scale model of interacting bacteria, but this approach lacks the mechanistic details available when using full enzyme constrained models (e.g. protein concentration predictions cannot be made [2]). This drawback reflects the lack of parameters endemic to the field when FBAwMC was introduced (*circa* 2007). More recently, quasi-resource allocation type constraints were added to community flux balance models through the incorporation of an L1 bound on total reaction flux in each community member [29]. This approach also eschews important details, like enzyme speed and size, which are both physiologically important attributes, figuring prominently in ec-FBA.

Community-simulating extensions of FBA [30, 31] typically assume that each community member grows at the same rate (otherwise the system is not at steady state), and metabolite flux exchange between members and their environments is weighted by the abundance of each microbe [26]. Previously, data scarcity prevented adding enzyme bounds to genome-scale metabolic models, but this problem has been attenuated by new *in silico* estimators, including machine learning [32], and omics-driven parameter estimation techniques [33], allowing for the parametrization of ec-FBA models at community scale.

[3] `https://bionumbers.hms.harvard.edu/bionumber.aspx?id=114971&ver=1&trm=ribosome+amino+acid&org=`

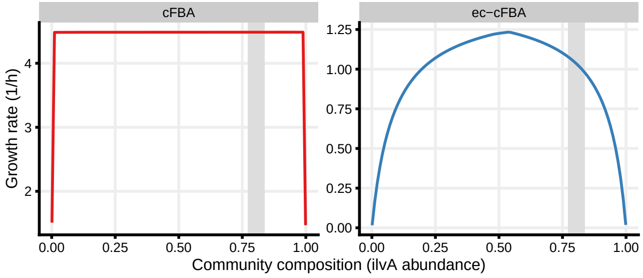

Figure S4: Comparison of growth predictions obtained for different compositions of ΔmetA–ΔilvA auxotroph *E. coli* community, using cFBA and ec-cFBA. In both simulations, the members were only allowed to exchange methionine and isoleucine. The vertical gray bar indicates the experimentally measured abundance [25].

We simulated mutually auxotrophic *E. coli* communities that were previously constructed experimentally by co-culturing mutants with complementary knockouts [24, 25]. Since each auxotrophic mutant lacks an essential gene for biosynthesis of a different amino acid [25], it is unable to grow in isolation, but may be rescued in a community where the amino acids may be exchanged with different mutants. However, the fundamental factors that determine the composition of the resultant communities are not well understood. Here, we hypothesize that each coupled community will adjust its abundance to grow as fast as possible, and investigate whether this optimality assumption holds by comparing measured abundance data to simulations using a classic cFBA, and enzyme-constrained cFBA (ec-cFBA).

First, we simulated a co-culture of ΔmetA (auxotrophic for methionine) and ΔilvA (auxotrophic for isoleucine) *E. coli* mutants. This pairing exhibits robust growth with a steady-state abundance of 20% ΔmetA, as established experimentally [25]. Supplementary Figure S4 compares the results obtained from ec-cFBA to conventional cFBA. Notably, with conventional cFBA the abundance of each member has only negligible effect on the community growth rate (up to extreme values), which is caused by the virtually identical metabolism of both members that can complement each other via zero-cost exchanges. This effect was previously prevented in simulations by incorporating non-mechanistic assumptions (i.e. a community MOMA-type simulation) that results in more realistic behavior [24]. In contrast, results from ec-cFBA clearly show two distinct growth regimes as the abundance of ΔmetA changes, with a better defined optimum at the intersection of the regimes. Each regime corresponded to a specific partner limiting the growth of the community. In this particular case, the biosynthesis cost of the biomass of each mutant differ because of both the knockout and the necessity to supply amino acids to other community members at a rate that satiates the abundance-controlled demand, which ultimately determines the optimal community composition.

Fascinatingly, when we extended the same analysis to all co-culture communities that demonstrated significant growth (Figure 1D in main text), we observed that ec-cFBA offers substantially improved predictions over cFBA. The comparison included only amino-acid pairings that exhibit significant growth in experimental conditions (over 10-fold biomass increase over inoculum) [25]. ec-cFBA provided better predictions than cFBA in all cases (measured via centered-log-ratio-transformed compositional distance), improving the correlation with experimental data from 0.197 (cFBA) to 0.453 (ec-cFBA). The ec-cFBA prediction compares well to the non-mechanistic (and non-steady state) approach used by Wintermute&Silver [24], who found a correlation of 0.42 across their dataset. Despite this, it is clear that both approaches are missing an important physiological constraint, since the predictive accuracy is relatively low. To improve, it might be necessary to incorporate either more precise regulatory effects or RBA-style constraints.

S3 Supporting results

Results from the simulation of 4-member community of *E. coli* mutants are summarized in Supplementary Figure S5.

Full results obtained from comparison of ec-FBA and sRBA are shown in Supplementary Figure S6. The reported results are a superset of ones in Figure 1B in main text.

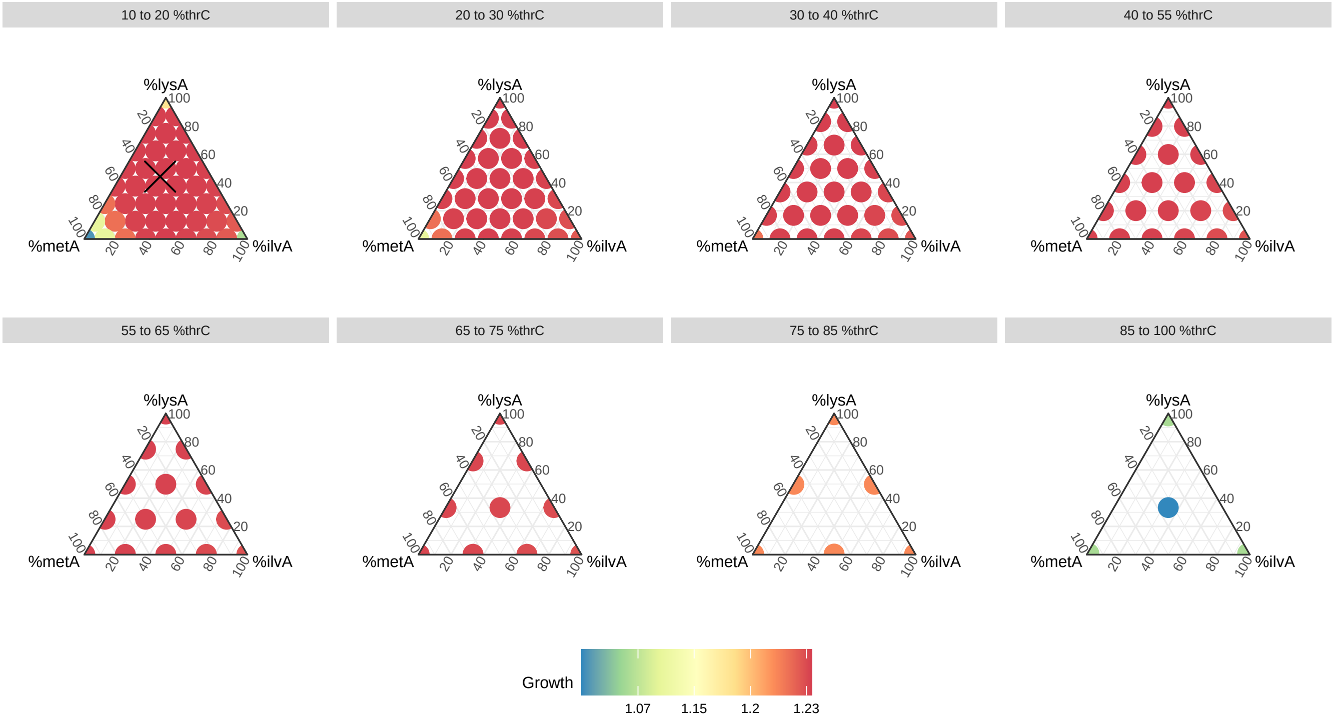

Figure S5: Enzyme kinetics constraints are not sufficient to robustly reproduce the composition of a 4-member auxotrophic community of *E. coli* mutants. The plot is organized as slices of a 3-dimensional Aitchison simplex of the community compositions [34]. Simulation of ΔmetA–ΔlysA–ΔilvA–ΔthrC community shows high variability in community compositions at near-optimal growth rates. The star represents the experimentally observed composition (ΔmetA 27%, ΔlysA 38%, ΔilvA 21%, ΔthrC 14%) [25].

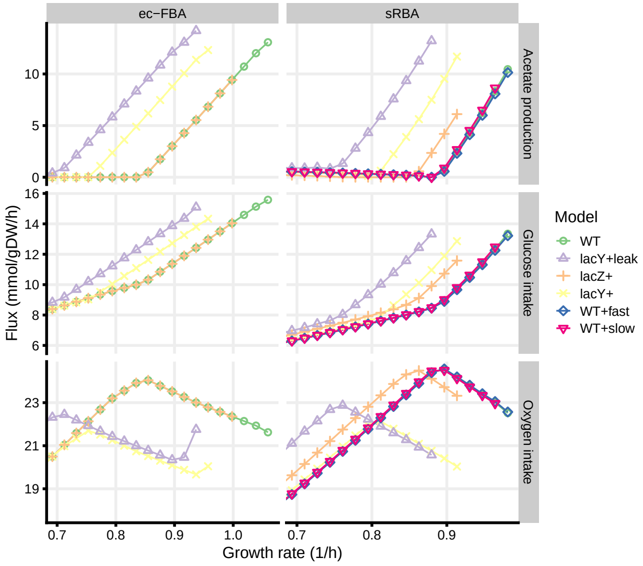

Figure S6: Comparison of results obtained from ec-FBA and sRBA models. The figure interpretation is the same as for Figure 1B in the main text, but includes results from additional simulations from a model where lacY is over-expressed but not leaking protons (labeled lacY+) and sRBA models where translation rate is changed from 12 to 9 and 17 amino acids per second (labeled respectively WT+slow and WT+fast, corresponding to the same labels in Figure 1C in the main text). Results for WT and lacZ+ almost completely overlap in the ec-FBA models; the WT model is able to grow approximately 5% faster. Similarly, results for WT, WT+slow and WT+fast almost completely overlap in the sRBA models; as the main difference, WT+fast is able to grow approximately 2% faster than WT, which itself grows around 2% faster than WT+slow.

S4 Constraint trees

ConstraintTrees.jl is available as a separate package that we implemented to provide the constraint-system representation for COBREXA 2. ConstraintTrees.jl is available from `https://github.com/COBREXA/ConstraintTrees.jl` and from Julia package repositories. Stand-alone tutorial documentation for ConstraintTrees.jl is available from `https://cobrexa.github.io/ConstraintTrees.jl/`.

Supplementary Table S1 provides an overview of the "grammar" of constraint system manipulations as implemented by ConstraintTrees.jl and COBREXA 2.

Constraint trees store a nested hierarchical structure of labeled constraints, organized into labeled directories. Each constraint consists of a value part and an optional bound part. Semantically, the value part defines a combination of variables in the system (typically a linear or quadratic one), and the bound part describes a condition that the value must satisfy (typically that the value of the combination of variables lies within a given interval). The labeled hierarchy carries no semantics in the constraint system, and serves only for manipulation convenience.

Shown in the Supplementary Table S1, the labeling and selection operations provide a systematic way to logically group any constraints, avoiding the need for name mangling (e.g., the oxygen exchange in organism 2, seen in the extension operation example, does not need to be labeled with unstructured identifier such as `"member2_R_EX_o2_e"` as common in other systems), and providing easy hierarchical access to all system constituents. Intersection and extension are the central operations, respectively representing intersection of feasible spaces of both constraint systems, and Cartesian product of the feasible spaces of constraint systems. The extension operation prevents any intersection of the variable sets of the given constraints, typically re-numbering the variable indexes of some of the operands, yielding a system where both original systems coexist independently. The interfacing operation is similar to extension, but additionally requires specification of the "module interfaces" (highlighted by arrows in the figure) which are used to connect the modules together (i.e., the systems are no longer independent in the result), and create an interface for the result that may be used for connecting more systems. Optimizing the system w.r.t. a given objective produces a "value tree" where constraints from the constraint tree are replaced by the evaluated combinations of the solved variables.

Notably, from the user perspective there is no difference between manipulating a constraint that holds a variable and a constraint that holds a linear combination (a "derived value") of the variables. This property has two main implications:

- It abstracts the user from having to manage variable vector allocations, instead the variables are typically allocated by using the extension (operator `+`) and interfacing (`join_interfaces`) operations.
- It enables transparent interfacing of constraint systems: For example, a system that represents a L2-parsimonious constraint can be built equivalently from the usual vector of flux-representing variables, and from a vector of variable combinations that derive the values from other contents of the model (such as sums of positive and negative reaction fluxes, and gene product capacity vectors in enzyme-constrained models).

Internally, the variable objects in the constraint solver are allocated implicitly, based on the presence of a referring index in the given constraint tree.

Operation name<br>`julia operation`	Example use		Example result
Constraining values<br>`Constraint`	`Constraint(` x_1 `,` ≥ 0 `)`	=	$x_1 \geq 0$
Labeling<br>`^`	`reactions^PFK^` $x_1 \geq 0$	=	**reactions**<br>PFK $x_1 \geq 0$
Selection<br>`.` `[]`	**exchanges**<br>O2 $x_{23} \in [-10, 10]$<br>`.exchanges.O2`	=	$x_{23} \in [-10, 10]$
Intersection<br>`*`	**stoichiometry**<br>O2_e $x_{23} - x_{85} = 0]$<br>`*`<br>**exchanges**<br>O2 $x_{85} \geq -10$	=	**stoichiometry**<br>O2_e $x_{23} - x_{85} = 0$<br>**exchanges**<br>O2 $x_{85} \geq -10$
Extension<br>`+`	**organism1**<br>O2_e $x_{23} - x_{85} = 0$<br>CO2_e $x_{15} - x_{68} = 0$<br>...<br>`+`<br>**organism2**<br>O2_e $x_{23} - x_{85} = 0$<br>CO2_e $x_{15} - x_{68} = 0$<br>...	=	**organism1**<br>O2_e $x_{23} - x_{85} = 0$<br>CO2_e $x_{15} - x_{68} = 0$<br>...<br>**organism2**<br>O2_e $x_{153} - x_{215} = 0$<br>CO2_e $x_{145} - x_{198} = 0$<br>...
Module interfacing<br>`interface_constraints`	`interf…(`<br>**organism1**<br>**stoichiometry**<br>O2_e $x_{23} - x_{85} = 0$<br>...<br>PFK KO $x_1 = 0$<br>**exchanges** ← interface<br>O2 $x_{85} \geq -10$<br>...<br>`,`<br>**organism2**<br>**stoichiometry**<br>O2_e $x_{23} - x_{85} = 0$<br>...<br>ACALD KO $x_2 = 0$<br>**exchanges** ← interface<br>O2 $x_{85} \geq -10$<br>...<br>`)`	=	**organism1**<br>**stoichiometry**<br>O2_e $x_{23} - x_{85} = 0$<br>...<br>PFK KO $x_1 = 0$<br>**exchanges**<br>O2 $x_{85} \geq -10$<br>...<br>**organism2**<br>**stoichiometry**<br>O2_e $x_{143} - x_{205} = 0$<br>...<br>ACALD KO $x_122 = 0$<br>**exchanges**<br>O2 $x_{205} \geq -10$<br>...<br>**interface_connection**<br>O2 $x_{85} + x_{205} - x_{251} = 0$<br>...<br>**exchanges** ← interface<br>O2 x_{251}<br>...
Optimization<br>`optimized_values`	`optimized_values(`<br>**organism**<br>**exchanges**<br>O2 $x_{85} \leq 0$<br>CO2 $x_{68} \geq 0$<br>biomass $x_{25} \geq 0$ ← maximize<br>...<br>`)`	=	**organism**<br>**exchanges**<br>O2 -5.2573<br>CO2 7.3622<br>biomass 0.873922<br>...

Table S1: Main operations on constraint trees illustrated on examples.

Bibliography

[1] Timo R Maarleveld, Meike T Wortel, Brett G Olivier, Bas Teusink, and Frank J Bruggeman. Interplay between constraints, objectives, and optimality for genome-scale stoichiometric models. *PLoS computational biology*, 11(4):e1004166, 2015.

[2] Benjamín J Sánchez, Cheng Zhang, Avlant Nilsson, Petri-Jaan Lahtvee, Eduard J Kerkhoven, and Jens Nielsen. Improving the phenotype predictions of a yeast genome-scale metabolic model by incorporating enzymatic constraints. *Molecular systems biology*, 13(8):935, 2017.

[3] Roi Adadi, Benjamin Volkmer, Ron Milo, Matthias Heinemann, and Tomer Shlomi. Prediction of microbial growth rate versus biomass yield by a metabolic network with kinetic parameters. *PLoS computational biology*, 8(7):e1002575, 2012.

[4] Pavlos Stephanos Bekiaris and Steffen Klamt. Automatic construction of metabolic models with enzyme constraints. *BMC bioinformatics*, 21:1–13, 2020.

[5] Douwe Molenaar, Rogier Van Berlo, Dick De Ridder, and Bas Teusink. Shifts in growth strategies reflect tradeoffs in cellular economics. *Molecular systems biology*, 5(1):323, 2009.

[6] Anne Goelzer, Vincent Fromion, and Gérard Scorletti. Cell design in bacteria as a convex optimization problem. *Automatica*, 47(6):1210–1218, 2011.

[7] Jonathan M Monk, Colton J Lloyd, Elizabeth Brunk, Nathan Mih, Anand Sastry, Zachary King, Rikiya Takeuchi, Wataru Nomura, Zhen Zhang, Hirotada Mori, et al. i ml1515, a knowledgebase that computes escherichia coli traits. *Nature biotechnology*, 35(10):904–908, 2017.

[8] Daan H De Groot, Julia Lischke, Riccardo Muolo, Robert Planqué, Frank J Bruggeman, and Bas Teusink. The common message of constraint-based optimization approaches: overflow metabolism is caused by two growth-limiting constraints. *Cellular and Molecular Life Sciences*, 77(3):441–453, 2020.

[9] David Heckmann, Anaamika Campeau, Colton J Lloyd, Patrick V Phaneuf, Ying Hefner, Marvic Carrillo-Terrazas, Adam M Feist, David J Gonzalez, and Bernhard O Palsson. Kinetic profiling of metabolic specialists demonstrates stability and consistency of in vivo enzyme turnover numbers. *Proceedings of the National Academy of Sciences*, 117(37):23182–23190, 2020.

[10] Michael Lynch and Georgi K Marinov. The bioenergetic costs of a gene. *Proceedings of the National Academy of Sciences*, 112(51):15690–15695, 2015.

[11] Markus Basan, Manlu Zhu, Xiongfeng Dai, Mya Warren, Daniel Sévin, Yi-Ping Wang, and Terence Hwa. Inflating bacterial cells by increased protein synthesis. *Molecular systems biology*, 11(10):836, 2015.

[12] Matthew Scott, Carl W Gunderson, Eduard M Mateescu, Zhongge Zhang, and Terence Hwa. Interdependence of cell growth and gene expression: origins and consequences. *Science*, 330(6007):1099–1102, 2010.

[13] Ron Milo. What is the total number of protein molecules per cell volume? a call to rethink some published values. *Bioessays*, 35(12):1050–1055, 2013.

[14] Evert Bosdriesz, Douwe Molenaar, Bas Teusink, and Frank J Bruggeman. How fast-growing bacteria robustly tune their ribosome concentration to approximate growth-rate maximization. *The FEBS journal*, 282(10):2029–2044, 2015.

[15] Markus Basan, Sheng Hui, Hiroyuki Okano, Zhongge Zhang, Yang Shen, James R Williamson, and Terence Hwa. Overflow metabolism in escherichia coli results from efficient proteome allocation. *Nature*, 528(7580):99–104, 2015.

[16] Alexander Schmidt, Karl Kochanowski, Silke Vedelaar, Erik Ahrné, Benjamin Volkmer, Luciano Callipo, Kevin Knoops, Manuel Bauer, Ruedi Aebersold, and Matthias Heinemann. The quantitative and condition-dependent escherichia coli proteome. *Nature biotechnology*, 34(1):104–110, 2016.

[17] Kai Zhuang, Goutham N Vemuri, and Radhakrishnan Mahadevan. Economics of membrane occupancy and respiro-fermentation. *Molecular systems biology*, 7(1):500, 2011.

[18] Mariola Szenk, Ken A Dill, and Adam MR de Graff. Why do fast-growing bacteria enter overflow metabolism? testing the membrane real estate hypothesis. *Cell systems*, 5(2):95–104, 2017.

[19] Matteo Mori, Terence Hwa, Olivier C Martin, Andrea De Martino, and Enzo Marinari. Constrained allocation flux balance analysis. *PLoS computational biology*, 12(6):e1004913, 2016.

[20] Samuel Wagner, Louise Baars, A Jimmy Ytterberg, Anja Klussmeier, Claudia S Wagner, Olof Nord, Per-Åke Nygren, Klaas J van Wijk, and Jan-Willem de Gier. Consequences of membrane protein overexpression in escherichia coli. *Molecular & Cellular Proteomics*, 6(9):1527–1550, 2007.

[21] UniProt Consortium. Uniprot: a worldwide hub of protein knowledge. *Nucleic acids research*, 47(D1):D506–D515, 2019.

[22] Birgit HM Meldal, Livia Perfetto, Colin Combe, Tiago Lubiana, João Vitor Ferreira Cavalcante, Hema Bye-A-Jee, Andra Waagmeester, Noemi Del-Toro, Anjali Shrivastava, Elisabeth Barrera, et al. Complex portal 2022: new curation frontiers. *Nucleic acids research*, 50(D1):D578–D586, 2022.

[23] Xiongfeng Dai, Manlu Zhu, Mya Warren, Rohan Balakrishnan, Vadim Patsalo, Hiroyuki Okano, James R Williamson, Kurt Fredrick, Yi-Ping Wang, and Terence Hwa. Reduction of translating ribosomes enables escherichia coli to maintain elongation rates during slow growth. *Nature microbiology*, 2(2):1–9, 2016.

[24] Edwin H Wintermute and Pamela A Silver. Emergent cooperation in microbial metabolism. *Molecular systems biology*, 6(1):407, 2010.

[25] Michael T Mee, James J Collins, George M Church, and Harris H Wang. Syntrophic exchange in synthetic microbial communities. *Proceedings of the National Academy of Sciences*, 111(20):E2149–E2156, 2014.

[26] Willi Gottstein, Brett G Olivier, Frank J Bruggeman, and Bas Teusink. Constraint-based stoichiometric modelling from single organisms to microbial communities. *Journal of the Royal Society Interface*, 13(124):20160627, 2016.

[27] Iván Domenzain, Benjamín Sánchez, Mihail Anton, Eduard J Kerkhoven, Aarón Millán-Oropeza, Céline Henry, Verena Siewers, John P Morrissey, Nikolaus Sonnenschein, and Jens Nielsen. Reconstruction of a catalogue of genome-scale metabolic models with enzymatic constraints using gecko 2.0. *Nature communications*, 13(1):3766, 2022.

[28] Qasim K Beg, Alexei Vazquez, Jason Ernst, Marcio A de Menezes, Ziv Bar-Joseph, A-L Barabási, and Zoltán N Oltvai. Intracellular crowding defines the mode and sequence of substrate uptake by escherichia coli and constrains its metabolic activity. *Proceedings of the National Academy of Sciences*, 104(31):12663–12668, 2007.

[29] Minsuk Kim, Jaeyun Sung, and Nicholas Chia. Resource-allocation constraint governs structure and function of microbial communities in metabolic modeling. *Metabolic Engineering*, 70:12–22, 2022.

[30] Siu Hung Joshua Chan, Margaret N Simons, and Costas D Maranas. Steadycom: predicting microbial abundances while ensuring community stability. *PLoS computational biology*, 13(5):e1005539, 2017.

[31] Ruchir A Khandelwal, Brett G Olivier, Wilfred FM Röling, Bas Teusink, and Frank J Bruggeman. Community flux balance analysis for microbial consortia at balanced growth. *PloS one*, 8(5):e64567, 2013.

[32] Alexander Kroll, Yvan Rousset, Xiao-Pan Hu, Nina A Liebrand, and Martin J Lercher. Turnover number predictions for kinetically uncharacterized enzymes using machine and deep learning. *Nature Communications*, 14(1):4139, 2023.

[33] St Elmo Wilken, Mathieu Besancon, Miroslav Kratochvíl, Chilperic Armel Foko Kuate, Christophe Trefois, Wei Gu, and Oliver Ebenhöh. Interrogating the effect of enzyme kinetics on metabolism using differentiable constraint-based models. *Metabolic engineering*, 74:72–82, 2022.

[34] Nicholas E. Hamilton and Michael Ferry. ggtern: Ternary diagrams using ggplot2. *Journal of Statistical Software, Code Snippets*, 87(3):1–17, 2018.
